# A Metabolic Storm: Clinical Warburg Effect in Poorly Differentiated Colorectal Adenocarcinoma

**DOI:** 10.7759/cureus.84806

**Published:** 2025-05-25

**Authors:** Yasmin Aly, Ahmed Abdelrahman, Kun Jiang, Marci Crowley, Enas Abdallah

**Affiliations:** 1 Internal Medicine, Faculty of Medicine, Alexandria University, Alexandria, EGY; 2 Diagnostic Imaging and Interventional Radiology, Moffitt Cancer Center, Tampa, USA; 3 Pathology, Moffitt Cancer Center, Tampa, USA; 4 Pathology, University of South Florida, Tampa, USA; 5 Malignant Hematology, Moffitt Cancer Center, Tampa, USA

**Keywords:** cancer metabolism, lactic acidosis, metastatic colorectal cancer, tumor induced hypoglycemia, warburg effect

## Abstract

Several theories have been proposed to explain the Warburg effect, a metabolic phenomenon increasingly recognized across both hematological and solid malignancies. We report a case in which both asymptomatic hypoglycemia and type B lactic acidosis developed a few months following the diagnosis of metastatic poorly differentiated colorectal adenocarcinoma. The patient had no symptoms despite having severely low blood glucose levels reaching the 20s (mg/dL), which could not be corrected by standard medical management and only responded to the chemotherapy regimen. The possible mechanisms underlying the hypoglycemia and lactic acidosis in the patient, the diagnostic criteria that define the Warburg effect and asymptomatic hypoglycemia, as well as the importance of exclusion of endocrinological disorders, sepsis, or ischemia, are discussed. This case highlights the significance of urgent diagnosis and management of this life-threatening metabolic disorder and raises awareness of this condition globally. Extensive research is required to determine the optimal therapeutic approach and to develop standardized guidelines for the management of such cases.

## Introduction

Lactic acidosis is commonly associated with critical conditions that lead to hypoxia and impaired systemic perfusion, such as heart failure, cardiac arrest, and sepsis [1]. However, oxygen depletion and hypoperfusion are not the only mechanisms behind lactic acidosis, as it can also occur under normal oxygen conditions [2-4]. Lactic acidosis has been reported in cases of malignancies, diabetic ketoacidosis, and alcohol intoxication, as well as with the use of medications including metformin, propofol, and antiretroviral therapy for HIV [1,5,6].

The Warburg effect is defined as excessive lactate production accompanied by hypoglycemia due to a rapid rate of glycolysis, despite the presence of functional mitochondria and normal oxygen levels (normoxia) [4,5]. Tumor cells tend to metabolize more glucose than normal tissue under aerobic conditions, due to a preferential metabolic shift toward glycolysis, which produces lactate instead of relying on oxidative phosphorylation [2]. This metabolic abnormality has been frequently reported in hematological malignancies (87%) but has also been observed in non-hematological malignancies (13%) [7], including solid tumors. Among solid tumors, metastatic small cell carcinoma and undifferentiated carcinoma have the highest number of case reports of the Warburg effect.

Colorectal cancer is the fourth most common cancer in the United States [1] and rarely presents with type B lactic acidosis. Very few case reports describe an association between the Warburg effect and colorectal cancer. Given the rarity of the association between the Warburg effect and colorectal cancer, as well as its emerging oncological significance, it is essential to diagnose and detect it early in cancer patients presenting with hypoglycemia and lactic acidosis. This report presents the case of a 67-year-old male patient diagnosed with metastatic poorly differentiated colorectal adenocarcinoma who presented with lactic acidosis and severe asymptomatic hypoglycemia three months after the diagnosis. We explain the pathophysiological mechanisms of lactic acidosis occurring alongside hypoglycemia in colorectal malignancies, compare outcomes from similar previously reported cases, and highlight the importance of prompt identification of this metabolic derangement to guide supportive care and inform therapeutic strategies.

## Case presentation

**Figure 1:**
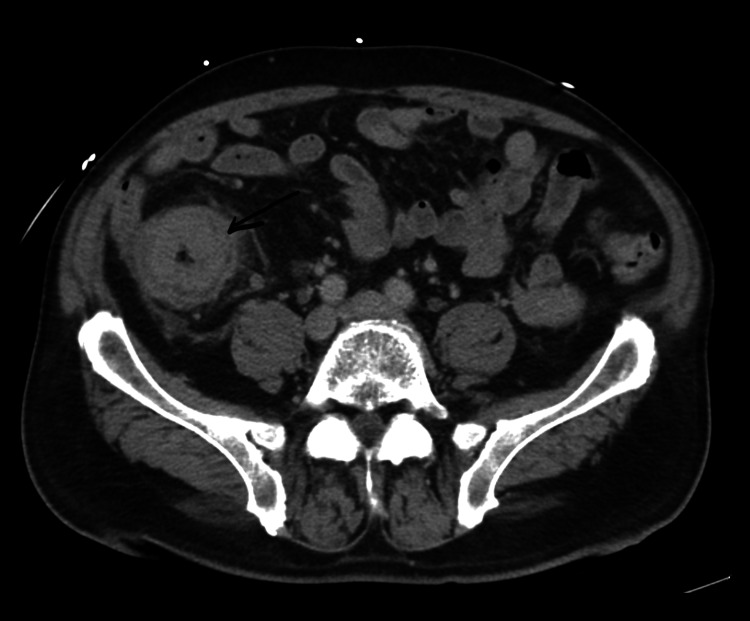
Contrast-enhanced CT of the lower abdomen shows circumferential wall thickening of the colon (black arrow), suspicious for primary site of colon cancer.

**Figure 2:**
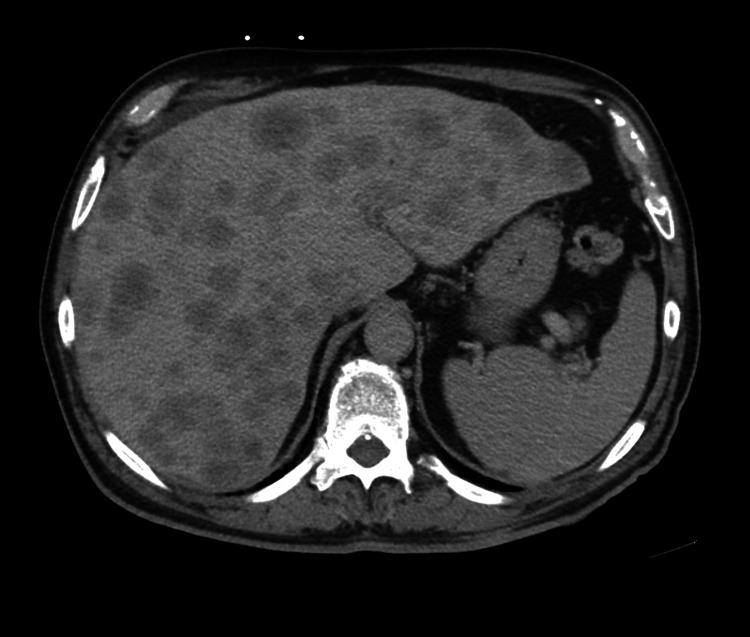
: Contrast-enhanced CT of the abdomen shows innumerable metastatic lesions in both lobes of the liver.

A 67-year-old male patient presented with a 30-pound unintentional weight loss over three months. He had a long-standing history of type 2 diabetes mellitus. CT abdomen and pelvis revealed an ascending colon mass (Figure 1), with multiple hepatic lesions (Figure 2), and FDG-PET/CT showed diffuse hypermetabolic activity in liver metastases and peritoneal implants. 

**Figure 3:**
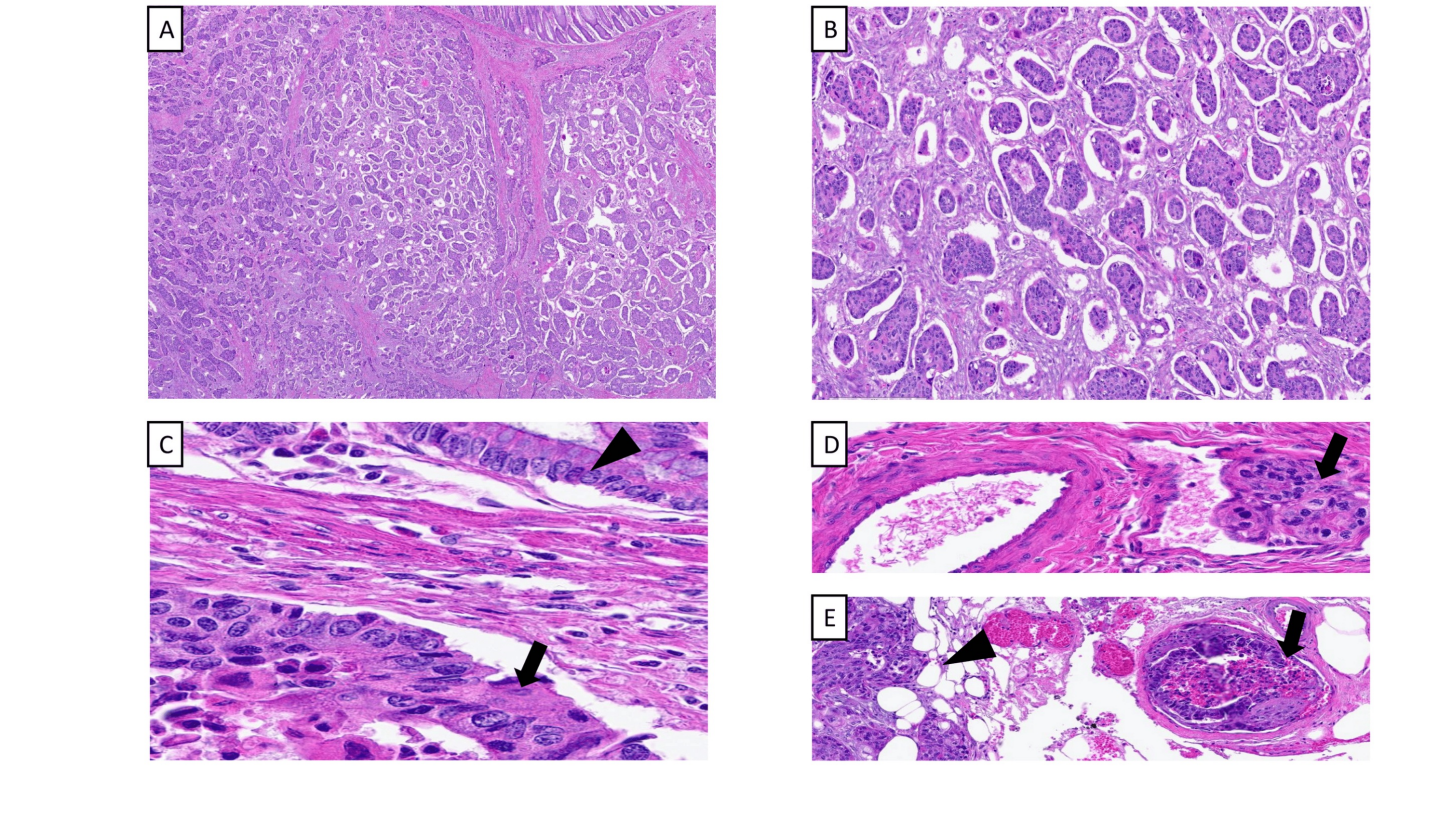
Histopathology of colorectal adenocarcinoma with prominent micropapillary features on hematoxylin and eosin (A, B): The classic morphology of micropapillary pattern is seen here with rounded small nests without a vascular core and abundant retraction artifact (× 20 and × 100, respectively); (C) Reverse polarity with the nuclei inward is seen in the neoplastic clusters (black arrow) compared to the normal polarity of the background colonic crypts (triangle) (× 20); (D, E): This aggressive subtype is associated with abundant lympho-vascular invasion (black arrows) and tumor deposits (triangle) (× 20 and × 10, respectively)

Colonoscopy for hematochezia identified a partially obstructing, oozing mass, and the patient underwent right hemicolectomy with resection of an 8 cm mid-ascending colon tumor. Pathology confirmed poorly differentiated adenocarcinoma (Figure 3) with T4aN2bM1a staging, extensive liver involvement, lymphovascular invasion, and negative margins. Molecular analysis identified *APC*, *TET2*, *MAP2K1*, and* TP53* mutations, the latter associated with poor prognosis and resistance to targeted therapies.

Three months after his diagnosis, the patient presented to our facility with lactic acidosis and hypoglycemia. On admission, physical examination was significant for cachexia, though vital signs remained stable. Initial laboratory investigations and venous blood gas analysis revealed profound hypoglycemia, severe lactic acidosis, high anion gap metabolic acidosis with a significant base deficit, mild transaminitis, elevated alkaline phosphatase (ALP), hypoalbuminemia, and markedly elevated carcinoembryonic antigen (CEA) levels (Table 1). Due to the patient’s severe lactic acidosis, computed tomography angiography (CTA) was done, and bowel ischemia was ruled out as a potential cause of lactic acidosis. 

**Table 1:** Laboratory results at admission INR: international normalised ratio NOTE: The most important parameters to diagnose the Warburg effect are highlighted in bold.

Parameters	Patient Values	Reference Ranges
Blood glucose level	55 mg/dL	70-110 mg/dl
Glycosylated hemoglobin	4.0 %	< 5.7%
Lactate level	15.4 mmol/L	< 2.2 mmol/L
Anion gap	18 mEq/L	4-12 mEq/L
PH	7.20	7.35-7.45
Bicarbonate (HCO₃⁻)	11.9 mmol/L	22-28 mmol/L
CO₂	16 mmol/L	23- 30 mmol/L
Base deficit	14.8 mmol/L	-2 to +2 mmol/L
WBC	15.03 × 10⁹/L	4.5 to 11.0 × 10^9^/L
Hemoglobin	11.6 g/dL	13.0- 18.0 g/dL
Platelet count	286 × 10⁹/L	150 to 400 × 10⁹/L
Alanine transaminase	28 U/L	7-56 U/L
Aspartate aminotransferase	154 U/L	8-33 U/L
Alkaline phosphatase	455 U/L	30-120 U/L
Albumin	2.4 g/dL	3.5-5.5 g/dL
Total Bilirubin	3.6 mg/dL	0.2-1.3 mg/dL
INR	2.0	0.8-1.2
Creatinine	0.4 mg/dl	0.7-1.3 mg/dL
Carcinoembryonic antigen	1960 ng/mL	< 2.5 mg/dL

Throughout his hospital stay, the patient’s blood glucose levels fluctuated between 30 and 40 mg/dL, yet he remained asymptomatic. A comprehensive endocrine panel was performed to assess for endocrinological or paraneoplastic causes of the patient's persistent hypoglycemia, revealing low levels of insulin, C-peptide, insulin-like growth factor (IGF)-2, and beta-hydroxybutyrate, with normal morning cortisol and adrenocorticotropic hormone (ACTH) (Table 2). 

**Table 2:** Comprehensive endocrine panel IGF: insulin-like growth factor; ACTH: adrenocorticotropic hormone NOTE: The most important parameters to diagnose the Warburg effect are highlighted in bold.

Parameters	Patient Values	Reference Ranges
Insulin	<2 µIU/mL	2.6-24.9 µIU/mL
Proinsulin	<2 pmol/L	3-20 pmol/L
C-peptide	0.1 ng/mL	0.5-2.0 ng/mL
IGF-1	28 ng/mL	115-307 ng/mL
IGF-2	138 ng/mL	333-967 ng/mL
Beta-hydroxybutyrate	1 mg/dL	< 3 mg/dL
Serum ketones	Negative	Below 0.6 mmol/L
Insulin antibodies	Undetectable	< 0.4 U/mL
Morning cortisol	20 µg/dL	5-25 µg/dL
ACTH	13 pg/mL	7.2-63.3 pg/mL

The patient was refractory to intravenous dextrose (including D10% and D20% infusion), so endocrinology was consulted. Steroid therapy was subsequently added to the treatment regimen, resulting in a mild increase in blood glucose levels. FOLFOX (leucovorin calcium (folinic acid), fluorouracil, and oxaliplatin) chemotherapy regimen was initiated less than 48 hours after admission, while the patient was still receiving steroid therapy. The patient's lactic acidosis and hypoglycemia were corrected following the initiation of chemotherapy. The patient elected to discontinue chemotherapy despite improvement in his metabolic profile and was ultimately discharged home with plans for hospice care. His blood glucose levels were in the 20s at home after discharge, and he had to eat a carbohydrate meal every one to two hours to maintain his blood glucose levels. Unfortunately, the patient died shortly while in hospice service.

## Discussion

Differential diagnosis of lactic acidosis

**Figure 4:**
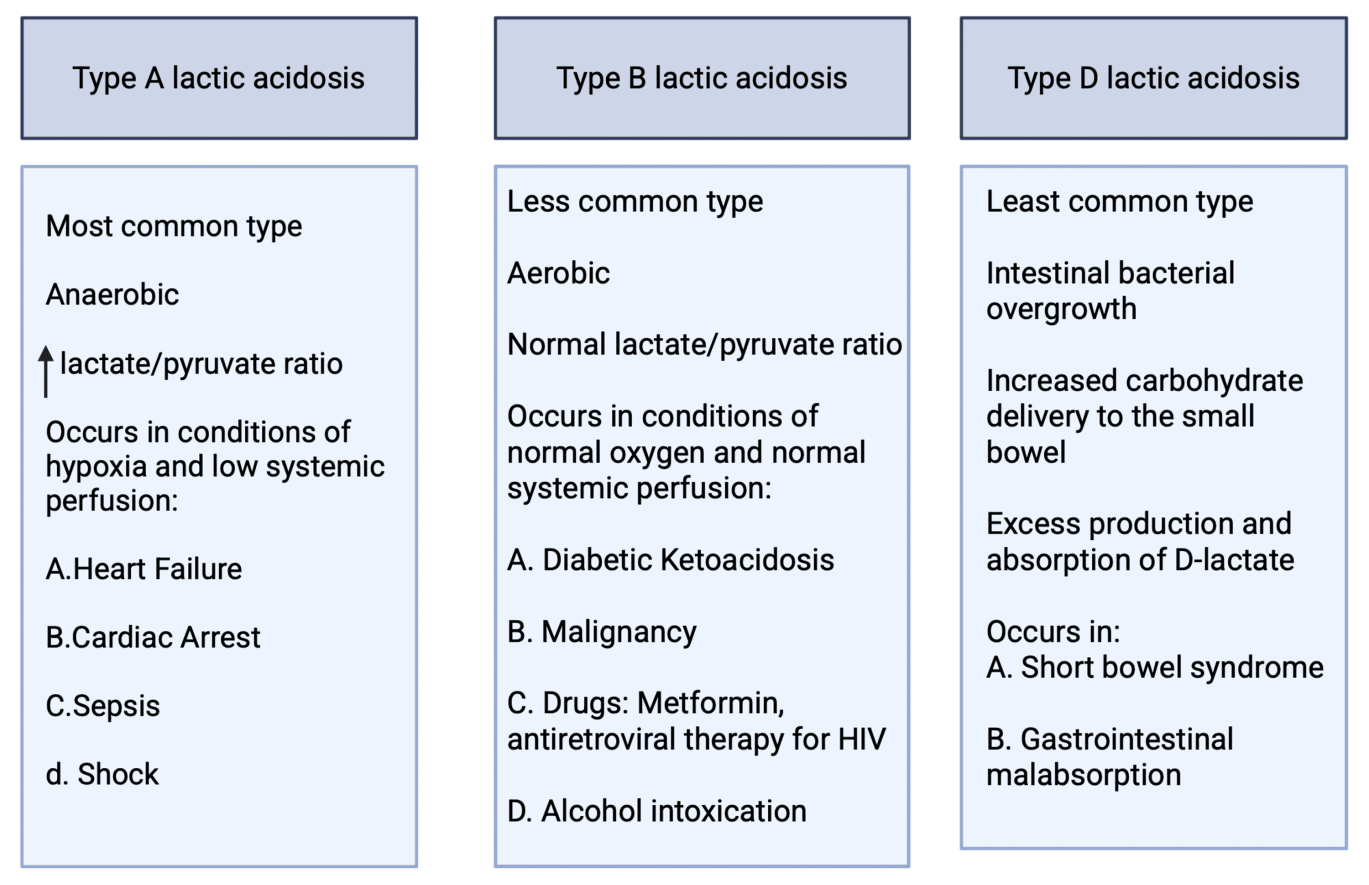
Types of hyperlactemia Created in biorender.com

Lactic acidosis can be classified into three types based on the underlying etiology (Figure 4). Type A lactic acidosis is the most prevalent form, typically arising in the context of poor oxygen supply and reduced systemic perfusion [1,6]. Type D lactic acidosis, although rare, may develop in patients with short bowel syndrome and gastrointestinal malabsorption. It is believed to result from intestinal bacterial overgrowth and increased carbohydrate delivery to the small bowel, leading to excess production and absorption of D-lactate. In contrast, type B lactic acidosis is less frequently encountered and has been increasingly associated with malignancies, particularly hematologic cancers [1,2,5].

More recently, there have been growing reports of type B lactic acidosis in solid malignancies, especially in cases of metastatic small cell lung cancers and undifferentiated carcinomas. The Warburg effect refers to the heightened glucose consumption observed in cancer cells, driven by an abnormally high rate of glycolysis despite the presence of oxygen and adequate systemic perfusion. This metabolic shift results in increased ATP production, hyperlacticemia, and is thought to contribute to rapid tumor progression. 

Diagnostic criteria

The Warburg effect can be diagnosed based on certain key criteria given in Table 2.

**Table 3:** Summary of key indicators for Warburg effect in metastatic colorectal cancer. FDG-PET: fludeoxyglucose-18 positron emission tomography

Test	Findings
Lactate levels	>5 mmol/L
Blood glucose levels	<60 mg/dL
pH	High anion gap metabolic acidosis
Ketone levels	Normal
FDG-PET	High uptake by tumor cells
Insulin/ C-peptide/IGF-2	Normal or low
Cortisol	Normal

Pathophysiology

There are several proposed mechanisms that can explain this effect. As for our case, it is believed that the overproduction of lactate, combined with decreased lactate excretion due to hepatic involvement by metastatic deposits, explains the high lactate levels recorded. The increased activity and overexpression of glycolytic enzymes by cancer cells, particularly type II hexokinase, can be attributed to the upregulation of hypoxia-inducible factor [3,8]. This occurs as the rapid growth of the tumor exceeds its vascular supply, creating a hypoxic microenvironment that favors anaerobic glycolysis. Additionally, it has been shown that type II hexokinase and other glycolytic enzymes are expressed in a concentration- and time-dependent manner by IGF-1 and insulin-binding proteins [9].

Cancer cells have the capacity to sequester thiamine pyrophosphate, a key cofactor of pyruvate dehydrogenase, leading to thiamine depletion and reduced acetyl-CoA production, which further promotes glycolysis while inhibiting the oxidative phosphorylation pathway [2]. This promotes lactic acidosis, diverts glucose into the pentose phosphate pathway to support rapid proliferation, and is further driven by upregulated glycolytic enzymes and increased expression of GLUT1 and GLUT3 transporters. This accelerated uptake contributes to hypoglycemia, concurrent with lactic acidosis resulting from the aerobic conversion of glucose to lactate.

In our case, the accelerated uptake and utilization of glucose by cancer cells overwhelmed the liver's ability to maintain glucose levels through gluconeogenesis and glycogenolysis, a capacity further diminished by metastatic infiltration of the liver. This clinically evident hypoglycemia, accompanied by excessive lactic acid production, characterizes a phenomenon known as “hyper-Warburgism” [10]. This metabolic state has been documented in cases of aggressive, large tumors with high glycolytic activity and metabolic demands, most notably in hepatocellular carcinoma, lymphoma, sarcoma, and metastatic colorectal cancer, as observed in our patient. 

Cancer cells rely on aerobic glycolysis, which increases their demand for glucose to produce an equivalent amount of energy and ATP compared to normal cells, ultimately leading to systemic glucose depletion in healthy cells [3]. In our case, it was hypothesized that neurons would be the most affected, potentially leading to severe neuroglycopenic symptoms. However, our patient remained asymptomatic even at glucose levels as low as the 20s mg/dL, suggesting that lactate acted as an alternative fuel for the brain, preserving its function and protecting against neurological symptoms typically associated with glucose deprivation. 

Several other mechanisms help explain systemic hypoglycemia seen in the Warburg effect, although they are not present in our case. Evidence suggests that high-molecular-weight IGF-2, secreted by non-islet cell tumors of mesenchymal origin, interacts with insulin and IGF-1 receptors, as well as binding proteins, to induce the expression of hexokinase and other glycolytic enzymes. This promotes increased peripheral glucose uptake and decreases hepatic glucose production, causing IGF-2-related non-islet cell tumor hypoglycemia (NICTH) [9]. In our patient, low insulin and IGF-2 levels, along with negative anti-insulin receptor autoantibodies, were documented, in contrast to patients with non-islet cell tumors, who typically exhibit elevated concentrations of IGFBP-1, IGFBP-2, and circulating free IGF-1 and IGF-2 [11-15]. This finding supports our hypothesis of excluding both insulin-mediated and paraneoplastic IGF-2-mediated glucose utilization as potential factors contributing to the low glucose levels in our patient [13]. 

Therapeutic approaches

Even though there is currently no known effective intervention for type B lactic acidosis and hypoglycemia, early detection of this metabolic disorder and timely initiation of appropriate chemotherapy have been proven to improve prognosis compared to delayed diagnosis and treatment [1]. In our case, the commencement of chemotherapy led to a remarkable reduction of lactic acidosis, resolution of hypoglycemia, and increased fludeoxyglucose-18 (FDG) uptake in the brain. Previous case reports have consistently considered factors such as the extent of hepatic dysfunction and metastatic involvement, the patient’s baseline metabolic profile, overall health status, and cancer stage, each of which may significantly influence the decision to initiate chemotherapy, given that some patients may be deemed unable to tolerate its potential complications [1,2]. Supportive care, including dextrose infusion, thiamine supplementation, bicarbonate therapy, and hemodialysis, has not conferred any survival benefit and has failed to correct either the hypoglycemia or the metabolic acidosis [7]. In fact, lactic acidosis may worsen with increased dextrose infusions, without a corresponding rise in glucose levels, as suggested by our proposed mechanism of glucose shunting toward cancer cells.

Prognosis

In general, the presence of lactic acidosis and hypoglycemia in a patient with advanced malignancy, in the absence of an endocrinological or other metabolic explanation, is indicative of a poor overall prognosis, with a reported mortality rate of approximately 77% within days to weeks [1], as documented in similar case reports (Table 3). 

**Table 4:** Reported cases of colorectal carcinoma that presented with Warburg effect in the past 15 years

Reference	Report year	Diagnosis	Age (years)	Sex	pH	Lactate (mmol/L)	Bicarbonate (mEq/L)	Liver Mets	Transaminitis	Treatment	Outcome
Curent case	2025	Poorly differentiated adenocarcinoma of colon	67	Male	7.20	15.4	11.9	Yes	Yes	IV dextrose, IV steroids, and chemotherapy	Patient elected to stop chemotherapy and died in hospice care shortly after admission
Yokokawa et al. [7]	2025	Primary Signet Ring cell carcinoma of colon	66	Female	7.38	7.5	17	No	No	IV fluids, IV antibiotics, IV insulin therapy, Nasogastric tube	Palliative chemotherapy and the patient died on the 5th day of admission
Cheleng et al. [4]	2024	Rectosigmoid colon cancer	52	Female	7.4	3.6		Yes	_	Left hemicolectomy	Patient passed away on the 8^th^ day after surgery
Amakiri et al. [5]	2024	Stage IV colon cancer	43	Male	7.26	16.52	9	Yes	Yes	IV antibiotics, Bicarbonate drip, and urgent haemodialysis	Discharged to palliative care)
Abaleka et al. [6]	2024	Stage IV invasive colon cancer of the rectosigmoid area	61	Male	7.2	13.7	5.9	Yes	Yes	IV fluids, IV Zosyn and Vancomycin, IV bicarbonate	Patient passed away on the 8^th^ day of admission
Uga et al. [3]	2019	Stage IV colon cancer	61	Female	_	6.4	20	Yes	Yes	IV saline, Zosyn, and Vancomycin	Discharged to hospice care on day 3
Gharia et al. [1]	2017	Metastatic adenocarcinoma of colon	64	Female	7.43	7.2	14	Yes	Yes	IV fluids and bicarbonate	Poor prognosis (days)
Espinoza and Venook [2]	2011	Invasive moderately differentiated adenocarcinoma	44	Female	7.24	> 11	7	Yes	Yes	FOLFOX chemotherapy	Death from progression of cancer

## Conclusions

This report described the clinical progression of metastatic undifferentiated adenocarcinoma of the colon, accompanied by severe hypoglycemia and lactic acidosis that manifested during the advanced stage of the cancer. This condition should be strongly considered in patients with metastatic solid tumors and hepatic infiltration who present with elevated lactate levels and hypoglycemia, particularly when no alternative etiologies or mechanisms can account for these metabolic abnormalities. It is imperative that further studies and randomized controlled trials be encouraged to define optimal therapeutic protocols for addressing this emerging oncological emergency.
